# Evaluation of *Saussurea costus* Nanoparticles in the Treatment of Periodontitis: Impact on NF-κB and TNF-α Expression

**DOI:** 10.1055/s-0045-1810069

**Published:** 2025-08-07

**Authors:** Azzahra Salsabila Adira Moelyanto, Ira Arundina, Theresia Indah Budhy, Meircurius Dwi Condro Surboyo, Aqsa Sjuhada Oki, Cheng Hwee Ming

**Affiliations:** 1 Magister Program for Dental Health Science, Faculty of Dental Medicine, Airlangga University, Surabaya, Indonesia; 2 Department of Oral Biology, Faculty of Dental Medicine, Universitas Airlangga, Surabaya, Indonesia; 3 Department of Oral Pathology and Maxillofacial, Faculty of Dental Medicine, Universitas Airlangga, Surabaya, Indonesia; 4 Department of Oral Medicine, Faculty of Dental Medicine, Universitas Airlangga, Surabaya, Indonesia; 5 Department Physiology, Faculty of Medicine. University of Malaya, Kuala Lumpur, Malaysia

**Keywords:** nanoparticles, *Saussurea costus*, NF-κB, TNF-α, inflammation

## Abstract

**Objectives:**

*Porphyromonas gingivalis* is a major contributing pathogen in periodontitis and triggers an inflammatory response that leads to tissue damage, primarily through the activation of proinflammatory cytokines such as nuclear factor kappa B (NF-κB) and tumor necrosis factor-α (TNF-α). *Saussurea costus* , a medicinal plant known for its anti-inflammatory properties, offers a potential alternative to prevent inflammation. This study explores the effects of *S. costus* nanoparticles on inflammation in periodontitis, specifically examining their ability to reduce the expression of NF-κB and TNF-α.

**Materials and Methods:**

Twenty male Wistar rats were injected *with P. gingivalis* into the central incisor region of the mandible to induce periodontitis. The rats were then treated with *S. costus* nanoparticles, alongside a control group. Immunohistochemical staining was performed to assess the expression of NF-κB and TNF-α at two time points: 2 and 5 days posttreatment. Bone and lower anterior gingival sulcus tissues were analyzed for immunohistochemical expression of these markers.

**Statistical Analysis:**

Statistical analysis was performed using the Kruskal–Wallis and Mann–Whitney U tests.

**Results:**

Treatment with *S. costus* nanoparticles resulted in significantly lower expressions of NF-κB and TNF-α compared with the control group at both 2 and 5 days posttreatment ( *p* < 0.05).

**Conclusion:**

The *S. costus* nanoparticles effectively reduced NF-κB and TNF-α expression in periodontitis, with a more pronounced effect observed on day 5 compared with day 2, suggesting its potential as a therapeutic agent in managing periodontal inflammation.

## Introduction

Periodontal disease is a chronic inflammatory condition affecting periodontal tissues, primarily seen in adults. Its prevalence is significant, with estimates indicating that 20 to 50% of the global population is affected. 1 In Indonesia, a 2018 survey reported a prevalence of periodontitis at 71.4%, with chronic periodontitis being the most common form, affecting approximately 50% of the adult population. 2 The primary etiological factor is the colonization of specific pathogenic bacteria in the oral cavity, notably *Porphyromonas gingivalis* , which is found in approximately 80.5% of cases. 3 *Porphyromonas gingivalis* can invade and adhere to gingival epithelial cells, 4 releasing several virulence factors, including lipopolysaccharides (LPS), enzymes (proteases and collagenases), capsules, and fimbriae. 5 These factors enhance bacterial colonization and promote coaggregation with other bacteria, exacerbating the inflammatory response.

LPS, a major contributor to inflammation, activates toll-like receptor 4 (TLR4), 6 which in turn stimulates the nuclear factor kappa B (NF-κB) signaling pathway, leading to increased transcription of proinflammatory cytokines, including interleukin (IL-1, IL-6, IL-8), and tumor necrosis factor α (TNF-α). 7 This inflammatory cascade accelerates periodontal tissue damage. Current therapies for chronic periodontitis primarily focus on plaque control through scaling and root planning. 8 Antibiotic therapy is often used to reduce *P. gingivalis* colonization 9 ; however, prolonged use can lead to bacterial resistance and adverse effects, such as alterations in oral flora, gastrointestinal disturbances, and reduced antibiotic efficacy.

Recent research has shifted toward natural alternatives for safer long-term treatment. *Saussurea costus* , a medicinal plant native to the Indian Himalayan region, has garnered attention for its anti-inflammatory and antimicrobial properties, attributed to compounds like dehydrocostus lactone and costunolide. 10 11 These compounds have demonstrated significant activity against both gram-positive and gram-negative bacteria, including *Staphylococcus aureus* 12 13 and *Pseudomonas aeruginosa* . 14 *Saussurea costus* exhibits antibacterial activity through several mechanisms primarily attributed to its rich content of bioactive compounds, including sesquiterpene lactones (e.g., costunolide and dehydrocostus lactone), flavonoids, and phenolic acids. 15 These constituents can disrupt bacterial cell membranes, leading to increased permeability and leakage of intracellular contents. Additionally, they interfere with bacterial DNA and RNA synthesis, hinder energy metabolism, and impair essential enzymatic functions. 15 These multimodal actions contribute to the plant's effectiveness against a wide range of gram-positive and gram-negative bacteria.

Despite the benefits of herbal extracts, their clinical effectiveness can be hindered by poor bioavailability. 16 Nanoparticle technology offers a solution by enhancing drug distribution, stability, and bioavailability while minimizing side effects. 17 The nanometer-sized particles (10–1,000 nm) can effectively penetrate cellular membranes, improving therapeutic outcomes. 18 Given the promising potential of *S. costus* in the nanoparticles, this study investigates their anti-inflammatory effects on periodontitis induced by *P. gingivalis* . We focus on the expression of NF-κB and TNF-α over 2 and 5 days, as these inflammatory biomarkers are crucial in mediating the inflammatory response associated with this condition.

## Materials and Methods

### Experimental Animals

In this study, the experimental subjects were male Wistar rats ( *Rattus norvegicus* ) aged 5 to 6 months, weighing between 250 and 300 g. The research protocol received ethical approval from the Institutional Ethics Committee, under registration number 0259/HRECC.FODM/III/2024.

### Periodontitis Model

A bacterial injection of *P. gingivalis* (Pg ATCC 33277) was administered at a concentration of 10 ^9^ CFU in 20 µL phosphate-buffered saline. 19 An injection of 0.03 mL was delivered using a 0.5-mL syringe into the gingival incisive sulcus of the mandible. The induction of *P. gingivalis* was performed every 3 days for 2 weeks, until clinical signs of bleeding on probing, redness, and gingival swelling were observed. Histological analysis was performed using hematoxylin and eosin staining to evaluate chronic inflammatory cell infiltration.

### Treatment with *Saussurea costus* Nanoparticles

The nanoparticles extracted from *S. costus* were produced based on the previous studies. 20 The size of the *S. costus* nanoparticles is 119.7 nm, and their polydispersity index is 0.182. 20 A total of 20 rats were employed and categorized into four groups based on treatment regimens and durations. Three experimental groups received nanoparticles extracted from *S. costus* , administered into the gingival sulcus using a 1-mL syringe (0.02 mL per application), while the control group received distilled water. The animals were sacrificed at two time points, after 2 and 5 days of treatment, respectively, to evaluate the time-dependent effects. During the treatment, the oropharyngeal regions of the experimental animals were covered with sterile gauze to prevent ingestion of the nanoparticle extract. This treatment was performed once daily for 2 and 5 days.

### Expression of NF-κB and TNF-α

Lower central incisors and alveolar bone tissue were collected following euthanasia by CO _2_ inhalation after 2 and 5 days of nanoparticle treatment. The tissue samples were fixed in 10% formalin for 24 hours and decalcified with ethylenediaminetetraacetic acid (EDTA) for 45 days. Immunohistochemical staining was performed using the standard streptavidin-biotin-peroxidase complex method to bind primary antibodies, utilizing the Universal LSAB2 System Kit. The primary antibodies used were NF-κB (anti-p65 antibody [nuclear factor-KB P65], polyclonal, antibody online GmbH, Germany) and TNF-α (anti-TNF-α antibody, polyclonal, antibody online GmbH, Germany). Expression levels were quantified by counting positive staining in the alveolar bone area in the central incisive mandibular (as a region of interest). All measurements were conducted using a light microscope (Nikon H600L, Nikon, Japan) with a magnification of 400X, examining five fields of view by a single blind operator.

### Statistical Analysis

The expression data for each parameter were presented as the mean ± standard deviation. Data normality was assessed using the Shapiro–Wilk test, and homogeneity was evaluated using the Levene test. Significance testing was performed using the Kruskal–Wallis test, and differences in NF-κB and TNF-α expression between days 2 and 5 were evaluated using the Mann–Whitney *U* test. A *p* -value of less than 0.05 was considered statistically significant for each group. Statistical analysis was conducted by one of the authors, who was blinded to the group allocations to reduce potential bias.

## Results

### Expression of NF-κB

**Fig. 1:**
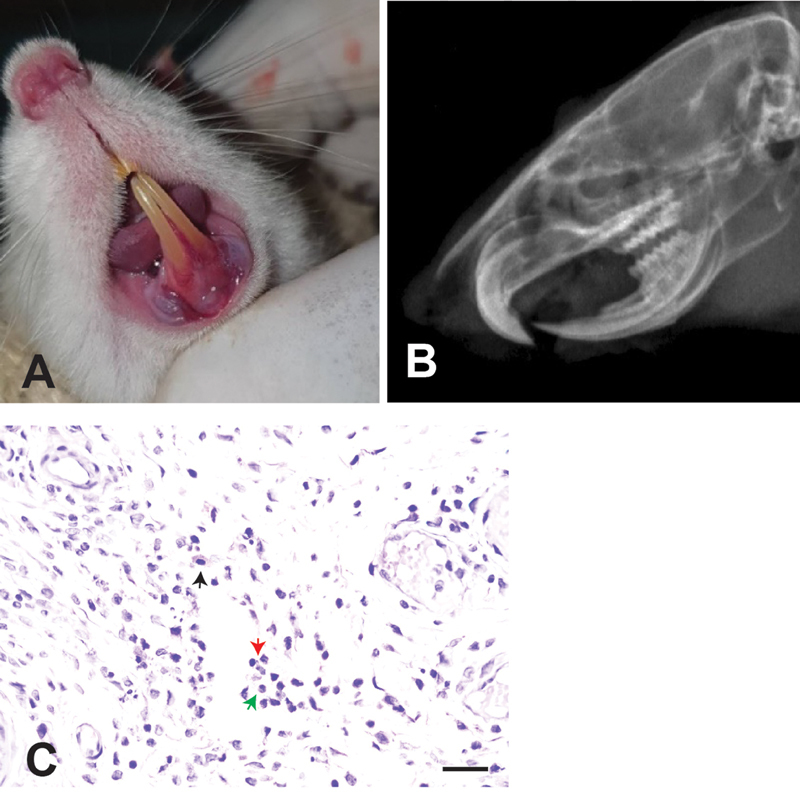
The clinical observation of lower mandibular incisive after 2 weeks of injection of *Porphyromonas gingivalis* . ( **A** ) Clinical signs of gingival inflammation; ( **B** ) decreased bone density, indicating resorption of alveolar bone; and ( **C** ) the presence of inflammatory cell in the periodontal tissue, macrophages ( *black arrow* ), neutrophils ( *red and green arrows* ). scale bar = 100 μm.

Following 2 weeks of *P. gingivalis* injection, the Wistar rats exhibited clinical signs of inflammation, including reddened gingiva, edema, gingival recession, and bleeding on probing ( Fig. 1A ). The radiographic analysis further indicated a decrease in bone density, evidenced by the appearance of gingival recession around the mandibular central incisors and radiolucent areas in the incisive region ( Fig. 1B ). Tissue section and staining using hematoxylin and eosin staining revealed signs of chronic inflammation characterized by the presence of lymphocytes, plasma cells, and macrophages in the alveolar bone ( Fig. 1C ). The Wistar rats developed significant clinical and histopathological signs of inflammation following the injection of *P. gingivalis* over 2 weeks.

**Fig. 2:**
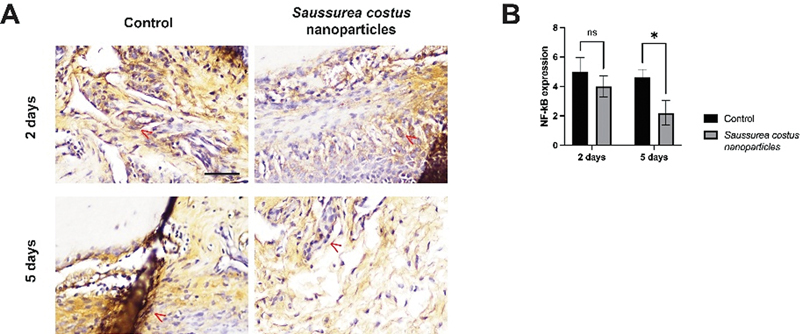
The treatment with *Saussurea costus* nanoparticles significantly reduced NF-κB expression in the alveolar bone. ( **A** ) Immunohistochemical staining for NF-κB expression. ( **B** ) Comparison of NF-κB expression levels after 2 and 5 days of treatment. * *p* < 0.05; ns = not significant; scale bar = 100 μm; *n* = 5. The *red arrowhead* indicates a cell-expressed NF-κB.

Two days after treatment with the *S. costus* nanoparticles (4.0 ± 0.707), there was no significant difference in the NF-κB expression compared with the control group (5.0 ± 0.987; *p* = 0.229). However, on day 5, the expression of NF-κB showed a significant reduction ( *p* = 0.001) in the alveolar bone of the lower central incisors, as shown in Fig. 2 . The treatment of *S. costus* nanoparticles significantly reduced the NF-κB expression (2.2 ± 0.837) in the alveolar bone of Wistar rats by day 5 posttreatment ( *p* = 0.001), indicating its potential anti-inflammatory effects.

### Expression of TNF-α

**Fig. 3:**
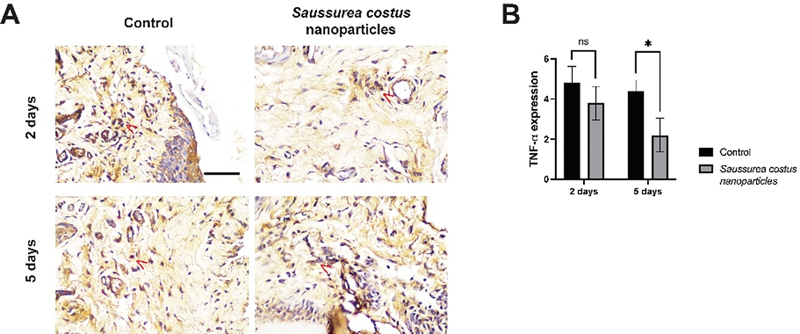
The treatment with *Saussurea costus* nanoparticles significantly reduced TNF-α expression in the alveolar bone. ( **A** ) Immunohistochemical staining for TNF-α. ( **B** ) Comparison of TNF-α expression levels after 2 and 5 days of treatment. * *p* < 0.05; ns = not significant; scale bar = 100 μm; *n* = 5. The *red arrowhead* indicates a cell-expressed NF-α.

Similarly, after 2 days of *S. costus* nanoparticle treatment (3.8 ± 0.837), there was no significant difference in TNF-α expression when compared with the control group (4.8 ± 0.837; *p* = 0.214). In contrast, on the fifth day posttreatment, the TNF-α expression demonstrated a significant reduction ( *p* = 0.002), as shown in Fig. 3 for the alveolar bone of the lower central incisors. The treatment with *S. costus* nanoparticles also resulted in a significant reduction of TNF-α expression in the alveolar bone by day 5 posttreatment (2.2 ± 0.837), contrasting with the lack of difference observed at day 2 ( *p* = 0.214). This finding, along with the significant decrease in NF-κB expression noted earlier, highlights the therapeutic potential of *S. costus* nanoparticles in mitigating inflammatory markers associated with periodontitis. The temporal reduction in both NF-κB and TNF-α suggests a cumulative anti-inflammatory effect, reinforcing the extract's role in managing periodontal inflammation.

## Discussion

Inhibition of NF-κB and TNF-α is crucial in the treatment of periodontitis for several reasons. First, NF-κB is a key transcription factor that regulates the expression of various proinflammatory cytokines, including TNF-α. 21 By inhibiting NF-κB, the inflammatory cascade is effectively dampened, reducing the overall inflammatory response that contributes to periodontal tissue destruction. 22 TNF-α, a potent proinflammatory cytokine, plays a central role in the pathogenesis of periodontitis by promoting inflammation, bone resorption, and tissue breakdown. High levels of TNF-α are associated with increased osteoclast activity, leading to alveolar bone loss—a hallmark of periodontitis. 23 Therefore, targeting TNF-α can help mitigate these destructive processes, preserving periodontal health. Moreover, controlling the inflammatory response is essential to restoring the balance between proinflammatory and anti-inflammatory factors in the periodontal environment. 24 This balance is vital for tissue healing and regeneration. By reducing the levels of NF-κB and TNF-α, treatments can promote a more favorable healing environment, potentially leading to improved clinical outcomes in patients with periodontitis. 25

*Saussurea costus* has garnered attention for its significant anti-inflammatory properties, making it a promising candidate for managing inflammatory conditions, including periodontitis. The plant contains bioactive compounds such as dehydrocostus lactone and costunolide, which have been shown to inhibit the production of proinflammatory cytokines and mediators. 11 The anti-inflammatory effects of *S. costus* are primarily attributed to its ability to modulate signaling pathways involved in inflammation. These compounds have been shown to inhibit nitric oxide (NO) production in LPS-induced mouse macrophage cells, a key pathway in inflammation. 26 For instance, it can inhibit the activation of NF-κB, a key regulator of inflammatory responses, thereby reducing the expression of various inflammatory cytokines, including TNF-α, IL-1, and IL-6. 27 By suppressing these cytokines, *S. costus* can help mitigate the chronic inflammation often observed in periodontal disease.

Furthermore, *S. costus* exhibits antioxidant properties that contribute to its anti-inflammatory effects. 28 By scavenging reactive oxygen species (ROS) and reducing oxidative stress, the extract can prevent cellular damage and further inflammatory responses. 29 30 This dual action—targeting both inflammatory pathways and oxidative stress—enhances its therapeutic potential. Additionally, the use of nanoparticles to deliver *S. costus* extract can improve its bioavailability and efficacy, allowing for targeted action at the site of inflammation. This delivery method maximizes the extract's anti-inflammatory effects while minimizing systemic side effects, making it a suitable option for long-term treatment.

In the clinical context, bacterial dysbiosis is a well-established trigger for periodontal inflammation, leading to persistent activation of NF-κB and elevated TNF-α levels in gingival tissues. 22 Periodontitis is often associated with a shift toward pathogenic bacterial communities, such as *P. gingivalis* and *Tannerella forsythia* , which activate host immune responses through LPS-TLR signaling, culminating in NF-κB-mediated transcription of proinflammatory cytokines. 31 Additionally, systemic conditions like diabetes mellitus further exacerbate periodontal inflammation by promoting a hyperinflammatory state and impaired resolution of inflammation, partly through sustained TNF-α expression. 32 By demonstrating the suppression of NF-κB and TNF-α, our findings suggest that *S. costus* , particularly in the nanoparticle form, may hold translational potential for managing periodontitis in patients with underlying systemic diseases or microbial imbalance, bridging the gap between preclinical findings and clinical applications.

## Conclusion

*Saussurea costus* nanoparticles effectively reduced NF-κB and TNF-α expression in periodontitis, with a more pronounced effect observed on day 5 compared with day 2, suggesting its potential as a therapeutic agent in managing periodontal inflammation. In conclusion, the anti-inflammatory properties of *S. costus* , supported by its bioactive compounds and enhanced by nanoparticle delivery, position it as a valuable therapeutic agent for managing inflammation in conditions like periodontitis. Further research into its mechanisms and clinical applications will help solidify its role in periodontal therapy.
